# Arf4 Determines Dentate Gyrus-Mediated Pattern Separation by Regulating Dendritic Spine Development

**DOI:** 10.1371/journal.pone.0046340

**Published:** 2012-09-25

**Authors:** Sachi Jain, Seo Yeon Yoon, Lei Zhu, Jens Brodbeck, Jessica Dai, David Walker, Yadong Huang

**Affiliations:** 1 Gladstone Institute of Neurological Disease, San Francisco, California, United States of America; 2 Gladstone Institute of Cardiovascular Disease, San Francisco, California, United States of America; 3 Biomedical Sciences Graduate Program, University of California San Francisco, San Francisco, California, United States of America; 4 Department of Neurology, University of California San Francisco, San Francisco, California, United States of America; 5 Department of Pathology, University of California San Francisco, San Francisco, California, United States of America; Massachusetts General Hospital, United States of America

## Abstract

The ability to distinguish between similar experiences is a critical feature of episodic memory and is primarily regulated by the dentate gyrus (DG) region of the hippocampus. However, the molecular mechanisms underlying such pattern separation tasks are poorly understood. We report a novel role for the small GTPase ADP ribosylation factor 4 (Arf4) in controlling pattern separation by regulating dendritic spine development. Arf4^+/−^ mice at 4–5 months of age display severe impairments in a pattern separation task, as well as significant dendritic spine loss and smaller miniature excitatory post-synaptic currents (mEPSCs) in granule cells of the DG. Arf4 knockdown also decreases spine density in primary neurons, whereas Arf4 overexpression promotes spine development. A constitutively active form of Arf4, Arf4-Q71L, promotes spine density to an even greater extent than wildtype Arf4, whereas the inactive Arf4-T31N mutant does not increase spine density relative to controls. Arf4′s effects on spine development are regulated by ASAP1, a GTPase-activating protein that modulates Arf4 GTPase activity. ASAP1 overexpression decreases spine density, and this effect is partially rescued by concomitant overexpression of wildtype Arf4 or Arf4-Q71L. In addition, Arf4 overexpression rescues spine loss in primary neurons from an Alzheimer's disease-related apolipoprotein (apo) E4 mouse model. Our findings suggest that Arf4 is a critical modulator of DG-mediated pattern separation by regulating dendritic spine development.

## Introduction

In the adult mammalian brain, the hippocampus plays a central role in the encoding and storage of certain types of memory, including spatial and episodic memory [1], [2]. The dentate gyrus (DG) subregion of the hippocampus mediates episodic memory formation and the disambiguation of similar but discrete events, a phenomenon known as pattern separation [3], [4], [5], [6]. Behavioral studies have shown that animals with impaired DG function are unable to differentiate between similar events or objects [7], [8], providing empirical evidence for a role of the DG in differentiating memories.

Like most excitatory neurons, granule cells of the DG are covered by dendritic spines that are important loci of synaptic plasticity [9], [10], [11], [12]. Spine morphology correlates with synaptic strength and structural plasticity; for instance, thin spines are highly motile and likely to respond to activity-induced changes, whereas mushroom spines have larger post-synaptic densities (PSDs) and are more stable [13], [14]. Aberrations in spine density and morphology are associated with a number of neurological disorders, including Alzheimer's disease (AD) [15], [16], [17]. In particularly, apolipoprotein (apo) E4–a major genetic risk factor for Alzheimer's disease–decreases spine density both *in vivo* [18], [19] and *in vitro* [20], [21], and is associated with spatial learning and memory impairments [22].

At the molecular level, small GTPases play a critical role in neuronal trafficking and spine morphogenesis [23], [24], [25], [26]. Members of the ADP-ribosylation factor (ARF) family of small GTPases and their regulators have gained increasing attention as important regulators of spine density and morphology [27], [28], [29]. Arf proteins are divided into three classes based on sequence homology: Class I (Arf1–3), Class II (Arf4–5), and Class III (Arf6). Like other small GTPases, Arfs act as molecular switches that cycle between an active, GTP-bound state and an inactive, GDP-bound state. This cycling activity is regulated by guanine nucleotide exchange factors (GEFs) and GTPase-activating proteins (GAPs) [30].

Previous studies have shown that Arf4 mRNA is highly expressed in the postnatal rat DG [31], suggesting that Arf4 might play a role in DG-associated learning and memory tasks. Furthermore, actin filament fragmenting was detected in the rod photoreceptors of transgenic *Xenopus laevis* expressing a mutant form of Arf4, indicating that Arf4 functions critically in actin cytoskeletal assembly [32]. Since dendritic spines are largely made up of actin, we considered the possibility that Arf4 might be involved in spine development.

Here, we report that Arf4^+/−^ mice display impairments in a DG-dependent pattern separation task, as well as significant spine loss and smaller mEPSCs in their DG granule cells. Consistent with our *in vivo* findings, knocking down Arf4 decreases spine density in primary neurons, whereas Arf4 overexpression significantly increases spine density. These effects are regulated by ASAP1, a GAP that was previously shown to form a complex with Arf4 and regulate GTP hydrolysis for Arf1, Arf4, and Arf5 [32], [33]. Furthermore, Arf4 overexpression restores spine loss in an AD-related apoE4 transgenic mouse model, suggesting a potential therapeutic use for Arf4.

## Materials and Methods

### Generation of Arf4^+/−^ mice

The Arf4^+/−^ mouse model was established based on an embryonic stem cell line from BayGenomics (CSH658). The ES cell line contains a gene trapping construct (pGT1lxf) in intron 1 of the Arf4 gene, located upstream of the gene encoding the β-galactosidase/neomycin-resistance fusion protein. The FastStart Taq DNA Polymerase dNTPack kit (Roche) was used to generate candidate forward primers designed for 200–500 base pair intervals of intron 1 of the Arf4 gene. One common reverse primer in the β-galactosidase reporter, RT416, was applied in all reactions (5′-GTCCTCTGGTGCTCAAAGACC-3′). Amplification with forward primer P8 (5′-TGGAAGCACAGGCCTTTAATCC-3′), located in intron 1, yielded a distinct PCR product of approximately 800 kb. PCR conditions were 34 cycles at 95°C for 30 s, 57°C for 30 s, and 72°C for 1 min, followed by a final extension at 72°C for 7 min. The PCR product was verified by sequencing.

The CSH658 ES cells were microinjected into C57Bl6 blastocysts to yield chimeras, which were identified by coat color. A chimeric male was crossed with wildtype C57Bl6 females, and germline transmission resulted in heterozygote males and females of the F1 generation. Mice were backcrossed to the C57Bl6 background for at least 5 generations for all studies, producing both Arf4^+/−^ and WT mice. C57Bl6 mice were purchased from the Jackson Laboratories. All animal procedures were approved by the Gladstone Institutes and the University of California San Francisco Animal Care and Use Committees.

### Genotyping of Arf4^+/−^ mice

Genotype of Arf4^+/−^ mice was determined by using 2 parallel PCRs. The first pair of primers consisted of the forward primer I8.1 (5′- AGCATATTCCCCTACTTAACTGTGTCTC-3′) and the reverse primer I8.1 Rev (5′-CAAAGGTGTTGCGGCACAGA-3′), both of which are in intron 1. The second pair of primers consisted of P8 and RT416, the same pair used to characterize the ES cell line. PCR conditions were as described for identification of the gene trap insertion site. DNA was prepared from 0.5 cm of cut tail from 21-day-old mice and digested overnight with sodium chloride-tris-EDTA buffer, followed by deactivation with Proteinase K. PCR products were electrophoresed on 2% agarose gels and stained with ethidium bromide.

### Preparation of mouse brain tissues and Neuro-2A cell lysates

Brains from WT or Arf4^+/−^ mice were collected after a 1 min transcardial perfusion with saline. The hippocampus and cortex were dissected from one hemibrain of each mouse and were homogenized with low detergent lysis buffer as previously described [34]. Samples were centrifuged at 35,000 rpm for 30 min at 4°C using a TLA 100.2 rotor of an Optima TL Ultracentrifuge (Beckman Coulter, Brea, CA) and the lysates were analyzed for Arf4 using western blot. Neuro-2A cells were lysed with low detergent buffer and centrifuged at 12,000 rpm for 10 min at 4°C, and supernatant was collected for western blot analysis. Rabbit anti-Arf4 was from Protein Tech Group (Chicago, IL), and rabbit anti-actin was from Sigma. Horseradish peroxidase-coupled anti-goat IgG was from Dako (Carpentaria, CA).

### Primary neuron preparation, transfection, and immunocytochemistry

Mixed hippocampal and cortical neuron cultures were prepared from E18–19 mice and grown on poly-L-lysine-coated coverslips as reported [20]. Neurons were fixed in PBS containing 4% paraformaldehyde, permeabilized with 0.1% Triton X-100, and blocked with 5% normal goat serum in PBS for 1 hour at room temperature. Neurons were incubated for one hour at room temperature with primary antibodies to goat HA (1∶500, Novus Biologicals, Littleton, CO), rabbit Flag (1∶500, Sigma), rabbit BDNF (1∶100, Santa Cruz Biotechnology), or mouse MAP2 (1∶500, Sigma). Secondary fluorophore-conjugated antibodies included donkey anti-goat Alexa488, donkey anti-goat Alexa594, donkey anti-goat Alexa647, donkey anti-mouse Alexa647, and donkey anti-rabbit Alexa594 (1∶1000, Invitrogen).

### Image analysis and quantification

For spine analysis of fluorescent cells, serial confocal images were taken with a BX60 BioRad Radiance 40X dry objective with a digital zoom factor of 2 for low magnification or of 4 for high magnification images. Z-stack sections were merged using LaserSharp2000 software. Neurons were selected randomly and 1 to 2 equivalent-length dendritic segments from each neuron were chosen for quantification of protrusions. Protrusion density and morphology were manually quantified using ImageJ software, according to criteria described [35].

### cDNA and small hairpin RNA constructs

HA-tagged human wildtype Arf4 was a gift from JD Lee (Scripps Research Institute, La Jolla, CA) [36] Arf4-HA point mutants (Arf4-HA-T31N and Arf4-HA-Q71L) were generated using the QuikChange II XL Site-Directed Mutagenesis kit (Strategene). mCherry-tagged human Arf4 was a gift from Paul Melancon (University of Alberta, Edmonton, Canada) [37]. The FUGW2-GFP and GFP-β-actin plasmids were gifts from Lennart Mucke and Steve Finkbeiner, respectively (Gladstone Institutes, San Francisco, CA) [13], [38]. Arf4-shRNA1 and Arf4-shRNA2 constructs were expressed under the U6 promoter using the FUGW2 vector. The target sequences used for the Arf4 shRNAs are as follows: 5′-TCTGGTAGATGAATTGAGA-3′ (Arf4-shRNA1) and 5′-AGATAGCAACGATCGTGAA-3′ (Arf4-shRNA2). Flag-tagged murine ASAP1 cDNA, a gift from Paul Randazzo (National Cancer Institute, Bethesda, MD), was expressed in the pCR2.1 vector [39].

### Staining of mouse brains

For β-galactosidase staining, brains from 4.5-month-old Arf4^+/−^ mice were removed, frozen in OCT compound, and sectioned at a thickness of 10 µm using a Leica CM1900 cryostat. Sections were washed 3 times in 0.02% Nonidet P-40/PBS, fixed in 4% paraformaldehyde in PBS for 10 min at room temperature, and stained in PBS with 5 mM K_3_Fe(CN)_6_, 5 mM K_4_Fe(CN)_6_, 2 mM MgCl_2_, 0.01% sodium deoxycholate, 0.02% Nonidet-P-40, and 1 mg/ml X-gal at 37°C for 16 h. After a series of ethanol washes, sections were cleared in xylene and mounted with Cytoseal.

For Golgi staining, 4.5-month-old Arf4^+/−^ and WT littermates were stained in parallel using modified Golgi-Cox impregnation of neurons following the manufacturer's protocol (FD NeuroTechnologies, Ellicott City, MD) (n = 4). Brains were sliced using a freezing-sliding microtome (Leica SM2000R) at a thickness of 150 µm. Images of the CA1 and DG were taken with a Leica CTR5000 brightfield 63X oil objective, coded, and analyzed in a blinded manner using ImageJ software.

For hematoxylin and eosin staining, following transcardial perfusion with saline, brains from WT and Arf4^+/−^ mice were fixed in 4% PFA-PBS for 48 hours, transferred to 70% ethanol, and embedded in paraffin. 5 µm sagittal sections were cut for conventional hematoxylin and eosin staining.

BDNF immunohistochemistry was performed on 30 µm coronal WT and Arf4^+/−^ brain sections following transcardial perfusion with saline and a 48 hour fixation in 4% PFA-PBS. Briefly, free-floating sections were treated with 3% H_2_O_2_ and 10% methanol in PBS to block endogenous peroxidase. The sections were incubated with PBS/10% normal donkey serum/1% milk/0.2% gelatin for 1 hour, followed by overnight incubation with primary anti-BDNF antibody (1∶100, Santa Cruz Biotechnology). Sections were further processed using a biotinylated secondary donkey anti-rabbit antibody (1∶1000, Jackson ImmunoResearch), avidin-peroxidase complex (ABC) and diaminobenzidine (DAB). The stained sections were mounted on slides, cleared with Xylene and coverslipped. Images were taken with a Leica CTR5000 brightfield 5X objective, and BDNF immunoreactivity was analyzed by densitometry using ImageJ software.

### Electrophysiology

Two-month old mice were deeply anesthetized and euthanized following UCSF animal protocol guidelines. Then the brains were quickly removed and immersed in ice-cold cutting solution containing (in mM) 234 sucrose, 2.5 KCl, 1.25 NaH2PO4, 10 MgSO4, 26 NaCO3, 11 glucose and 1.3 ascorbic acid, and oxygenated with 95% O_2_/5% CO_2_. Transverse slices of 325 µm were cut on a Leica VS1000 vibroslicer (Leica, Germany) and incubated at 32°C for 30 min in an interface incubation chamber (Automated Scientific, CA), after which the slices continued to be incubated in the same chamber at room temperature. For recording, slices were transferred to a submerged recording chamber and continuously perfused with oxygenated artificial cerebrospinal fluid (aCSF) containing (in mM) 126 NaCl, 2.5 KCl, 1.25 NaH2PO4, 1 MgSO4, 26 NaCO3, 10 glucose and 2 CaCl2 at 3 mL/min (25°C). Whole-cell recordings were performed on visually identified dentate granule cells and fully matured granule cells were identified by input resistance (less than 400 MΩ). To isolate miniature excitatory postsynaptic currents (mEPSCs), 100 µM picrotoxin(Sigma), 5 µM bicuculline (Tocris) and 0.5 µM tetrodotoxin (Abcam) were added to the perfusate. The internal pipette solution contains (in mM) 120 CsMeSO3, 4 NaCl, 2 MgCl2, 10 HEPES, 5 EGTA, 5 MgATP, 0.3 Na_3_GTP and 5 QX-314. Series resistance (<30 MΩ) was constantly monitored and the recording was discarded if changes >15% occur. Data were digitized at 20 kHz by a Multiclamp 700A amplifier (Axon Instruments, Union City, CA) and acquired with a Digidata-1322A digitizer and WinLTP program (WinLTP Inc, University of Bristol, UK). Offline analysis was performed using Mini-analysis program (Synaptosoft inc) and the threshold setting for event detection was set at 4x the amplitude of baseline noise. Four hundred events were analyzed for each cell and only events recorded 10 minutes after whole cell break-in were included in the data analysis.

### Behavioral tests

Behavioral testing was performed using male and female mice that were 4–5 months of age at the time of testing. All experiments and analyses were performed blind to genotype. The pattern separation test is used to measure an animal's ability to distinguish between similar events [40]. Mice were habituated to the pattern separation testing room for one hour prior to training. During the training period, mice were placed in an open chamber with a specific floor pattern and two identical objects, and were allowed to explore for 10 minutes. Following a 30-minute inter-trial interval, mice were placed in a second open chamber with a different floor pattern and two identical objects unique from the objects in the first trial. After 3 hours, mice were tested for 10 minutes in a chamber consisting of a floor pattern from either trial one or trial two, one object from trial one, and one object from trial two. The time each mouse spent exploring the object in the novel context (e.g., object from trial one in context from trial two) was compared with the time spent exploring the object in the old context. Exploration of an object was defined as the length of time a mouse's nose was 1 cm away from the object, and video recordings of the trials and test period were used to manually analyze exploration time.

For the Morris water maze test, the water maze pool (122 cm, diameter) was filled with opaque water (21°C) and contained a submerged platform (10 cm, diameter) during hidden trials [22], [34]. The ability of mice to locate the hidden platform was determined in two sessions (3.5 hours apart) per day for 5 days. Each session consisted of two 60 sec trials with a 15 min intertrial interval. The platform location remained constant during the hidden trials, and entry points were changed for each trial. The latency to reach the hidden platform was recorded as a measure of spatial learning. Probe trials (60 sec, platform removed) were performed 24, 72, and 120 hours after the hidden trials. Memory retention was measured by the percent time spent in the target quadrant compared to the average time spent in the other three quadrants, as well as by the number of crossings over the original position of the target platform compared to the number of crossings over the equivalent platform positions in other quadrants. Following the probe trials, the ability of mice to locate a clearly visible platform was tested in three sessions (two trials/session) to exclude differences in vision and swim speed. Performance was monitored with an EthoVision video-tracking system (Noldus Information Technology).

The open field test is a standard test for general locomotor activity, willingness to explore, and anxiety [41]. It consists of a square enclosure in which infrared detectors track animal movement. Locomotor and exploratory activity is assessed by the number of basic movements and rearings, whereas the proportion of time spent in the center of the enclosure is used as a measure of anxiety [42]. Mice were placed in the center of the chamber and were tested for 15 min.

The rotarod test, using a steady-speed rotarod set at 16RPM, was performed by placing mice on rotating drums and measuring each animal's latency to fall over a period of 300 seconds [43].

The Elevated Plus Maze is a test for rodent anxiety and is based on a rodent's aversion to open spaces [44]. The apparatus consists of two open arms and two enclosed arms at right angles to each other. Mice were placed on the central platform and were allowed to explore the apparatus for 10 minutes. Anxiety was assessed by comparing the amount of time spent in open versus enclosed arms.

### Statistical analysis

Unless stated otherwise, all values are expressed as mean±SEM. Statistical analyses were performed with GraphPad Prism software. Differences between the means were assessed by *t*-test or one factor ANOVA followed by a Bonferroni *post-hoc* test. A *p*-value of <0.05 was considered to be statistically significant. Statistical values are denoted as follows: * *p*<0.05, ** *p*<0.01, *** *p*<0.001.

## Results

### Arf4^+/−^ mice have impairments in a DG-dependent pattern separation task

**Figure 1:**
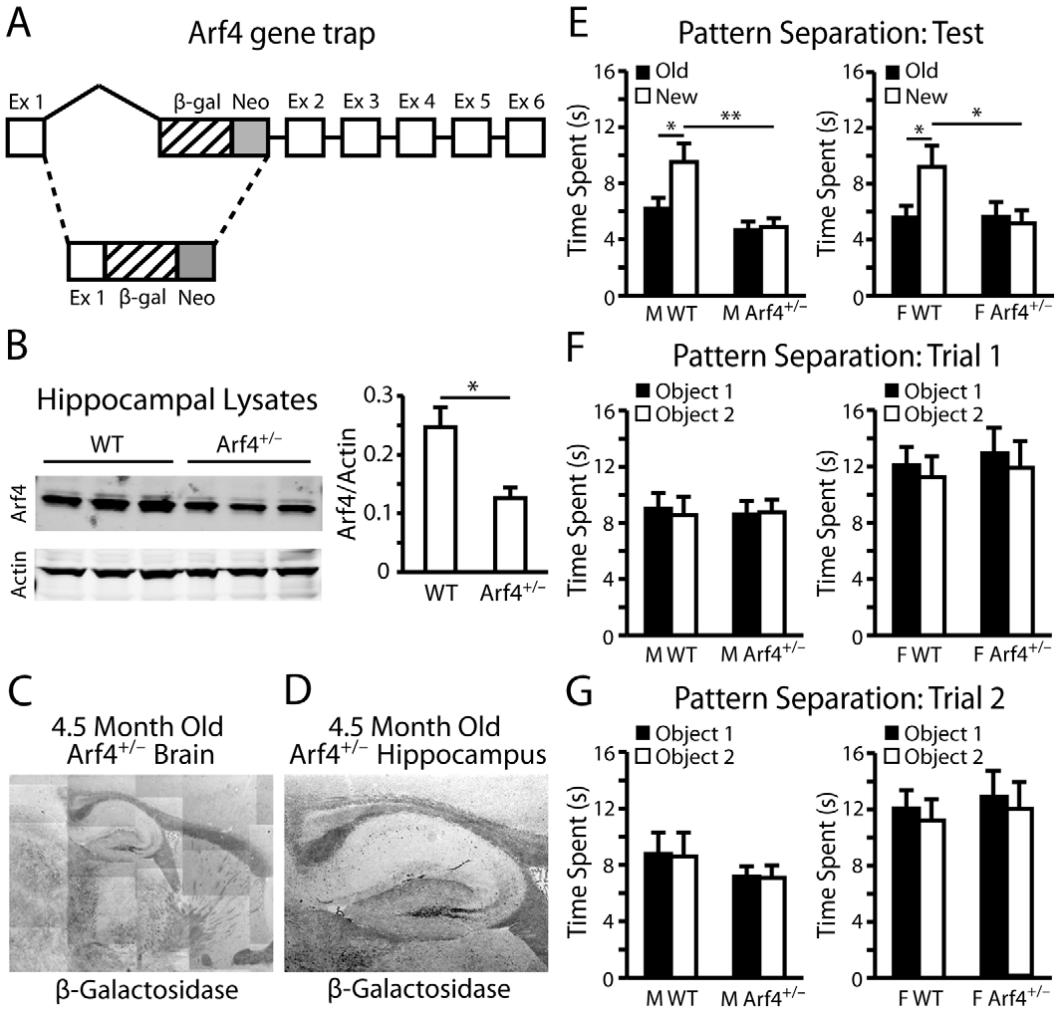
Arf4^+/−^ mice are impaired in a dentate-gyrus dependent pattern separation task. All mice were between 4–5 months of age at the time of experimentation. (*A*) A map of the Arf4 gene trapping construct obtained from BayGenomics (ES cell line CSH658). Ex, exon; β-gal, β-galactosidase gene; neo, neomycin-resistance gene. (*B*) Hippocampi from three WT and Arf4^+/−^ littermate pairs were homogenized, followed by immunoblotting with anti-Arf4 (left panel). Actin loading controls are shown. Quantification of Arf4 protein levels in hippocampi prepared from WT or Arf4^+/−^ mice (right panel). Arf4 protein levels were normalized to actin. (*C*) X-gal stained sagittal brain section from an Arf4^+/−^ mouse at 5X magnification. (*D*) Representative image from a 4.5-month-old Arf4^+/−^ hippocampus. (*E*) Quantification of the amount of time male (left panel) or female (right panel) WT and Arf4^+/−^ mice spent exploring a novel object/context during a pattern separation task. (N = 12–13 mice per genotype per sex). (*F*–*G*) Quantification of the amount of time male (left panel) or female (right panel) WT and Arf4^+/−^ mice spent exploring an object in a specific context during the first (*F*) or second (*G*) trial of the pattern separation task. All data are mean±SEM. * p<0.05, ** p<0.01.

To study the roles of Arf4 *in vivo*, we used a gene-trapping strategy to try to generate Arf4^−/−^ mice (Fig. 1A). Since Arf4^−/−^ mice were embryonically lethal, we focused our *in vivo* studies on Arf4^+/−^ mice. Arf4^+/−^ mice were fertile, viable and showed no overt phenotype. Arf4 protein levels were reduced by 49% in the hippocampus of Arf4^+/−^ mice compared to wildtype (WT) littermates (Fig. 1B). X-gal staining of 4.5-month-old Arf4^+/−^ mice showed that Arf4 is highly expressed in the DG (Figs. 1C and D), prompting us to investigate the potential roles of Arf4 in DG-dependent memory tasks.

**Figure 2:**
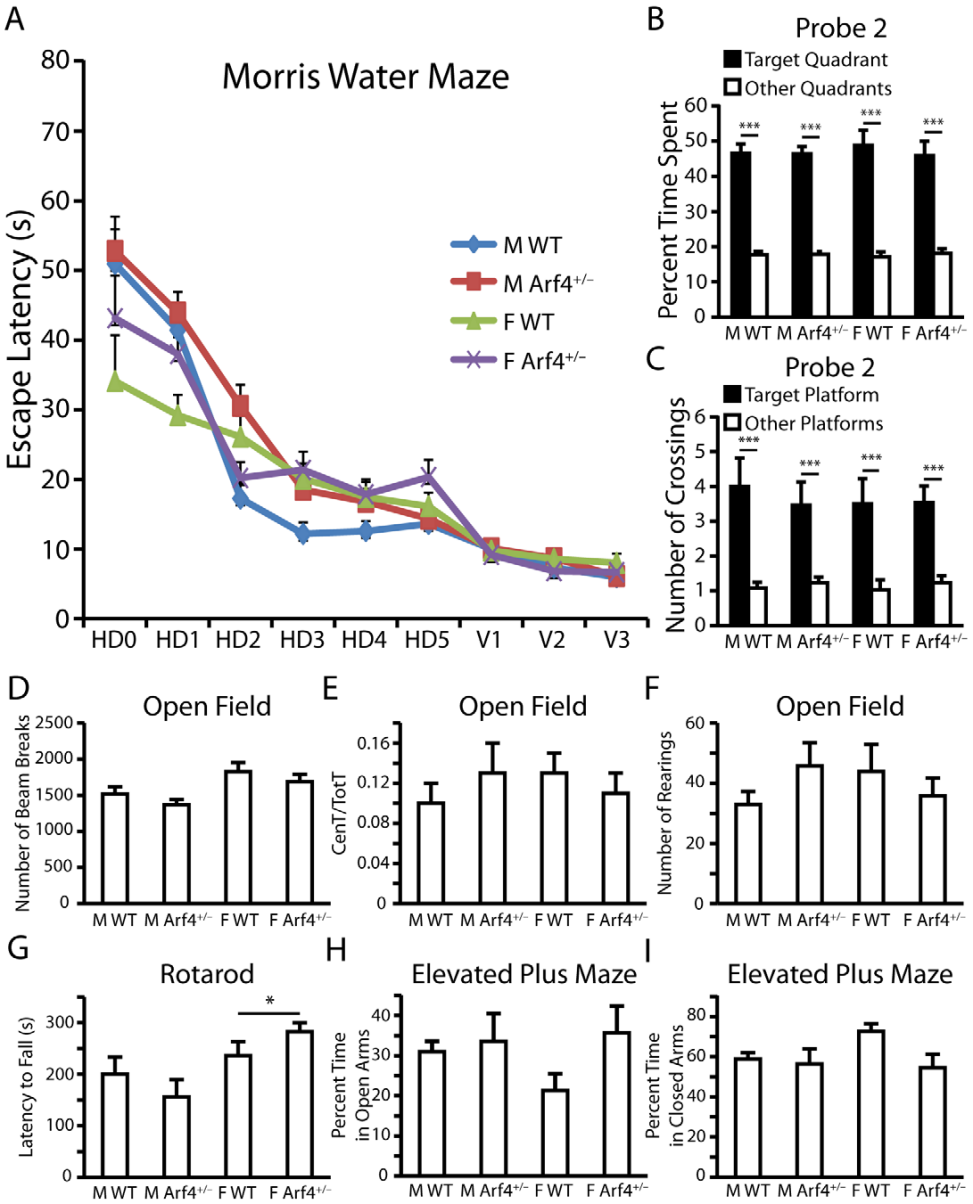
Arf4^+/−^ mice are not impaired in spatial learning and memory, general locomotor activity, motor coordination, or anxiety behavior. Mice were 4–5 months of age at the time of experimentation (12–13 mice per genotype per sex). (*A*) Results from Morris water maze test for spatial learning and memory. Graph of escape latency times. Points represent averages of daily trials. HD, hidden platform day (2 trials/session, 2 sessions/day); HD0, first trial on HD1; V, visible platform day (2 trials/session, 2 sessions/day). (*B–C*) Results from probe trials performed 72 h after the final hidden platform training (Probe 2). Data are presented as percent time spent in the target quadrant versus the average time spent in the other quadrants (*B*) and number of crossings over the original position of the target platform compared to the number of crossings over the equivalent platform positions in other quadrants (*C*). (*D–F*) Results from open field test for general locomotor activity. Data are presented as the total number of infrared beam breaks over the 15-minute testing period (*D*), ratio of activity in the center of the open field compared to activity in the center plus the periphery (*E*), and total number of rearings over a 15 minute period (*F*). CenT, total number of beam breaks in the center; TotT, total number of beam breaks in the center plus periphery. (*G*) Rotarod test for motor coordination. Rotarod was set at 16RPM and animals were tested during three independent trials, each lasting a maximum of 300 seconds. The average latency to fall is shown over all three trials. (*H–I*) Results from elevated plus maze test for anxiety. Maze consists of two open arms and two closed arms. The percent time spent by male and female WT and Arf4^+/−^ mice in the open (*H*) and closed (*I*) arms is shown. All data are mean±SEM. * p<0.05, *** p<0.001.

Since the DG is known to be involved in an animal's ability to distinguish between similar events [3], we asked whether the loss of one copy of Arf4 might affect performance in a pattern separation task. This task involved training mice to associate a certain environmental context with specific objects in two training sessions, followed by a testing session in which the rodents' ability to recognize context-object distinctions was analyzed. Whereas WT mice spent a greater proportion of time exploring the object in the novel context than the object in the familiar context, Arf4^+/−^ mice showed no difference in the proportion of time spent with either object during the testing phase (Fig. 1E). Neither WT nor Arf4^+/−^ mice showed significant differences in the amount of time spent with the two identical objects in either of the training sessions (Figs. 1F and G). Thus, Arf4^+/−^ mice were unable to effectively distinguish between two similar but unique situations. Interestingly, Arf4^+/−^ mice did not show deficits in spatial learning (Fig. 2A) or memory retention (Figs. 2B and C), suggesting that the neurological impairments are specific to DG-dependent pattern separation. Arf4^+/−^ mice were also not impaired in locomotor and exploratory activity (open field test) (Figs. 2D–F), motor coordination (rotarod) (Fig. 2G), or anxiety-related behaviors (elevated plus maze) (Figs. 2H and I).

### Reduced spine density and mEPSC amplitude in Arf4^+/−^ DG granule cells

**Figure 3:**
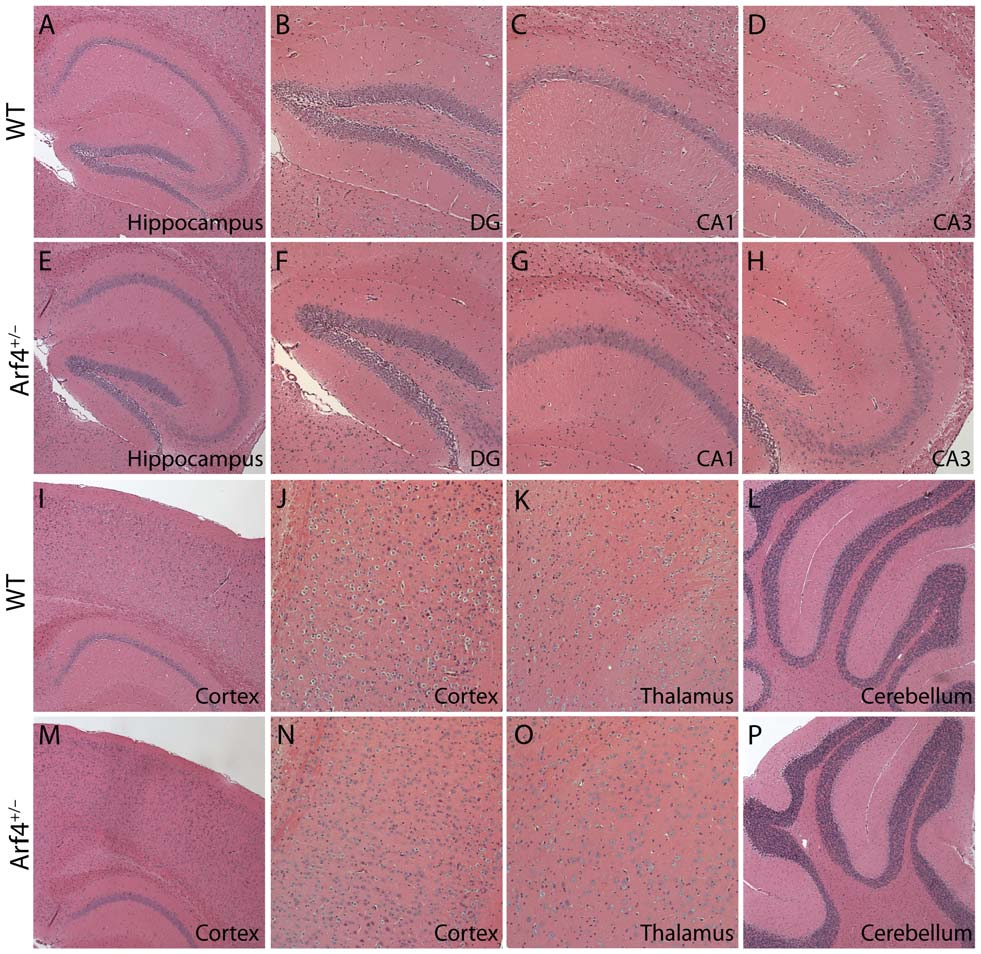
Overall structure and morphology of Arf4^+/−^ brains are not altered compared to WT controls. (*A–P*) Hematoxylin and eosin-stained paraffin brain sections from WT and Arf4^+/−^ mice. Representative images of the hippocampus (*A, E*), DG (*B, F*), CA1 (*C, G*), CA3 (*D, H*), cortex (*I, J, M, N*), thalamus (*K, O*), and cerebellum (*L, P*) from WT and Arf4^+/−^ mice are shown.

**Figure 4:**
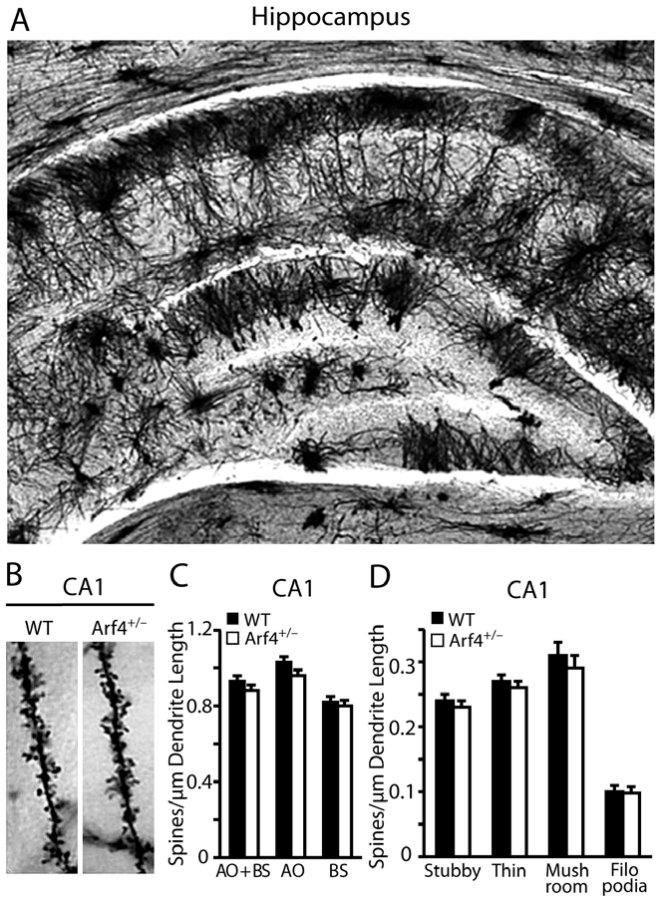
CA1 spine density is not altered in Arf4^+/−^ mice compared to WT mice. (*A*) Golgi impregnation of a WT hippocampus at 5X. (*B*) Representative dendrites for CA1 pyramidal neurons of WT and Arf4^+/−^ mice at 63X magnification. (*C*) Averaged total spine density in the CA1 region of WT and Arf4^+/−^ mice (28–30 neurons/genotype). (*D*) Spine densities for specific spine subtypes in the CA1 region of WT and Arf4^+/−^ mice.

**Figure 5:**
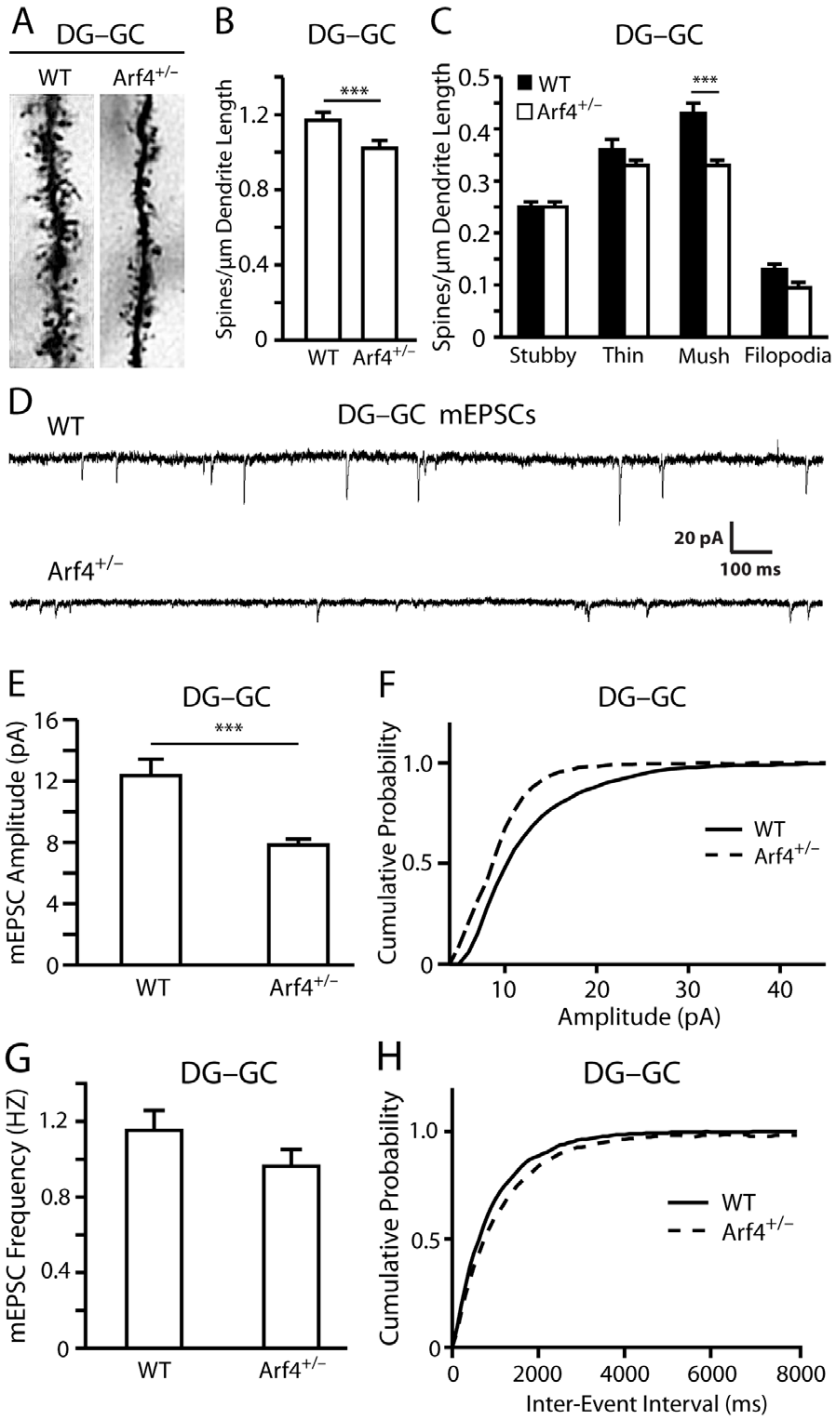
Decreased dendritic spine density and mEPSC amplitude in the DG of Arf4^+/−^ mice compared to WT mice. (*A*) Representative dendrites for DG granule cells of WT and Arf4^+/−^ mice at 4 months of age (n = 4 mice/genotype). (*B*) Averaged total spine density per µm dendrite length in the dentate gyrus of WT and Arf4^+/−^ mice (28–30 neurons/genotype). (*C*) Spine densities for specific spine subtypes in the DG of WT and Arf4^+/−^ mice. (*D–F*) Electrophysiological recordings reveal a decrease in amplitude of mEPSCs in Arf4^+/−^ (n = 9 cells) compared to WT controls (n = 6 cells) at 2 months of age. All data are mean±SEM. * p<0.05, ** p<0.01, *** p<0.001. (*G*–*H*) Spontaneous mEPSC frequency is not altered in Arf4^+/−^ granule cells (n = 9 cells) compared to WT granule cells (n = 6 cells) at 2 months of age.

The overall structures and morphologies of the hippocampus, cortex, and other brain regions appeared normal in Arf4^+/−^ brains compared to controls, as determined by hematoxylin and eosin staining (Fig. 3). We then examined the potential effects of Arf4 heterozygosity on neuronal fine structure using a modified Golgi-Cox staining protocol (Figs. 4A, 4B, and 5A). Both apical and basal dendrites of pyramidal neurons from the CA1 region (Fig. 4C), as well as dendrites from granule cells of the DG region (Fig. 5B), were analyzed (n = 4 mice/genotype). Although the CA1 region of Arf4^+/−^ mice did not show spine density (Figs. 4B, C) or morphology (Fig. 4D) alterations compared with controls, there was a significant decrease in total spine density in the granule cells of the DG (Figs. 5A and B), as well as a decrease in mushroom spine density (Fig. 5C), which is in line with the high level expression of Arf4 in the DG (Figs. 1C and D). Furthermore, the amplitude of miniature excitatory postsynaptic currents (mEPSCs) was 37% lower in Arf4^+/−^ DG granule cells than controls (Figs. 5D–F). The frequency of mEPSCs did not differ between the two genotypes (Figs. 5G and H). Thus, reducing Arf4 by 50% significantly impaired spine development and the electrophysiological function of granule cells in the DG.

### Arf4 is expressed in neurons and localizes to dendritic spines

**Figure 6:**
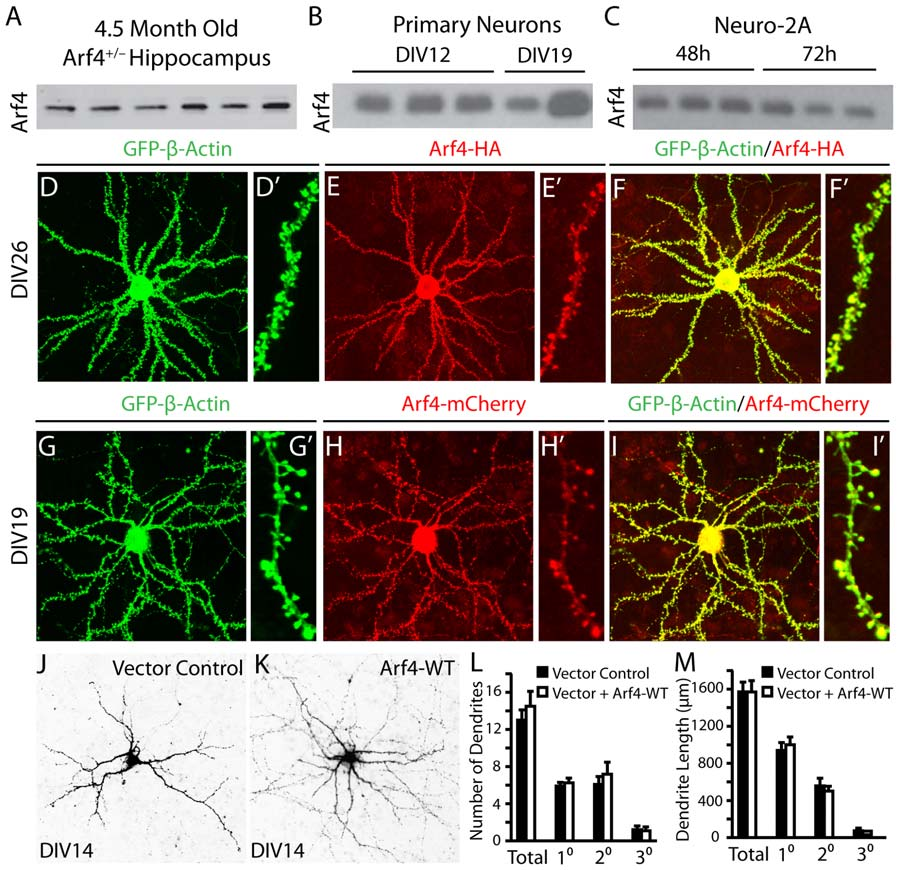
Expression of Arf4 in brain of 4.5-month-old mice and in primary neurons, and the effect of Arf4 overexpression on dendritic morphology. (*A*) Hippocampi from WT mice were prepared as described in methods, followed by immunoblotting with anti-Arf4 (n = 6). (*B*) Mixed hippocampal and cortical neurons were prepared from E18–E19 WT mouse embryos, and neuronal lysates were collected at DIV12 and DIV19. Samples were immunoblotted with anti-Arf4. (*C*) Lysates from WT Neuro-2A cells were collected 48 and 72 hours after plating, followed by immunoblotting with anti-Arf4. (*D–I′*) Primary neuron co-transfected with GFP-β-actin and Arf4-HA (*D–F′*) or Arf4-mCherry (*G–I′*) at DIV5 and imaged at DIV14. Higher (*D–I*) and lower (*D′– I′*) magnification images are shown. *(J, K)* Representative images of dendrite morphology for neurons transfected with a control vector (*J*) or vector plus Arf4-WT (*K*). (*L, M*) The number (*L*) and length (*M*) of primary, secondary, and tertiary dendrites were quantified. (n = 12 neurons per experimental condition). All data are mean±SEM.

Arf4 is expressed in the hippocampi of 4.5-month-old mice, as well as in primary neurons and Neuro-2a (N2a) neuroblastoma cells (Figs. 6A–C), as determined by western blots. When a HA- (Figs. 6D–F) or mCherry-tagged (Figs. 6G–I) form of human Arf4 was expressed in mouse primary neurons, Arf4 was visible throughout the soma and dendrites, including dendritic spines. Furthermore, both Arf4-HA and Arf4-mCherry co-localized with GFP-β-actin (Figs. 6D–I), which forms networks in dendritic spines [10]. These results point to a potential role for Arf4 in modulating neuronal function at the level of dendritic spines.

### Arf4 promotes dendritic spine development in primary neuronal cultures

**Figure 7:**
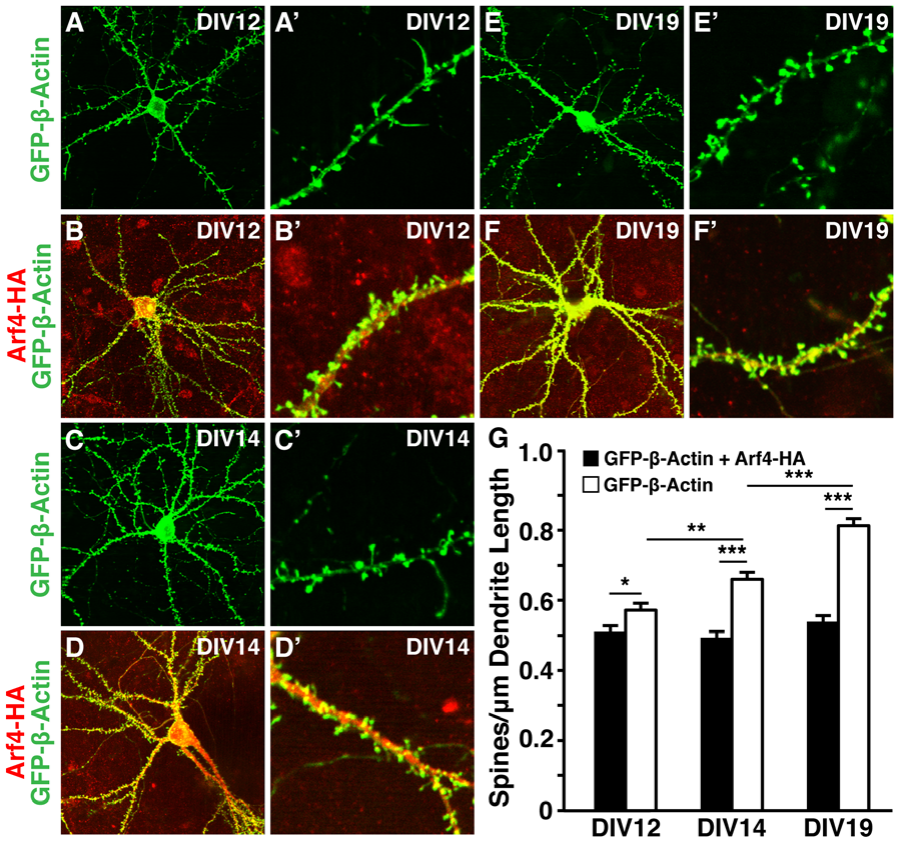
Arf4 overexpression promotes dendritic spine development in mouse primary neurons. (*A–B′*) Neurons transfected with GFP-β-actin alone (*A–A′*) or together with Arf4-HA (*B–B′*) at DIV5 and imaged at DIV12 (n = 9–10 neurons). (*C–D′*) Neurons transfected with GFP-β-actin alone (*C–C′*) or together with Arf4-HA (*D–D′*) at DIV5 and imaged at DIV14 (n = 8–10 neurons). (*E–F′*) Neurons transfected with GFP-β-actin alone (*E–E′*) or together with Arf4-HA (*F–F′*) at DIV5 and imaged at DIV19 (n = 8–9 neurons). (*G*) Averaged spine density of neurons transfected with GFP-β-actin alone or with GFP-β-actin plus Arf4-HA at several time points. All data are mean±SEM. * p<0.05, ** p<0.01, *** p<0.001.

To examine whether Arf4 regulates spine development, we transfected cultured primary mouse cortical and hippocampal neurons at 5 days *in vitro* (DIV5) with cDNA constructs encoding either human Arf4-HA and GFP-β-actin or GFP-β-actin alone. Overexpression of GFP-β-actin does not impair neuronal function or dendritic spine morphology and density and can, therefore, be used to highlight dendritic spines for imaging and quantification [29], [45]. Overexpression of Arf4 did not significantly alter total dendrite number or length, nor did it change the number or length of primary, secondary, or tertiary dendrites (Figs. 6J–M). However, Arf4 overexpression dramatically increased dendritic spine density at DIV12 (Figs. 7B and B′), DIV14 (Figs. 7D and D′), and DIV19 (Figs. 7F and F′) compared with control neurons at each time point (Figs. 7A, C, and E). Furthermore, this increase in spine density was significant across time points, whereas spine densities remained relatively constant throughout development for neurons transfected with GFP-β-actin alone (Fig. 7G). We observed similar increases in spine density in GFP-β-actin co-transfection experiments using Arf4-mCherry in the place of Arf4-HA.

**Figure 8:**
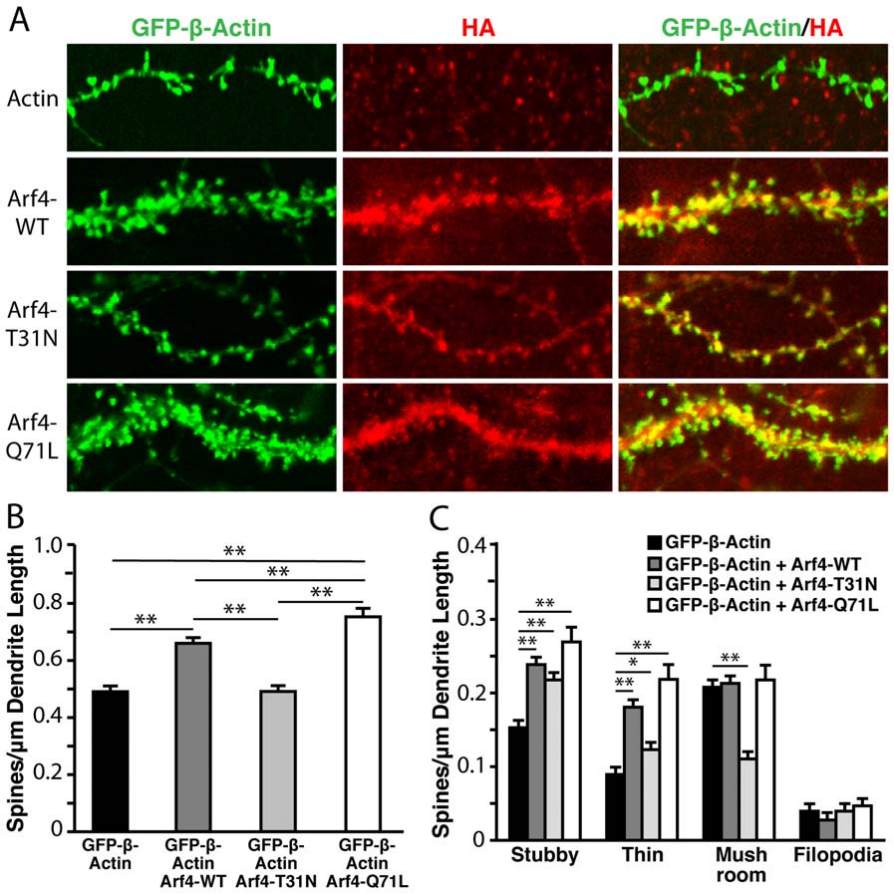
Overexpression of constitutively active Arf4 (Arf4-Q71L) promotes dendritic spine development to a greater extent that Arf4-WT. (A) Primary hippocampal and cortical neurons were cotransfected at DIV5 with GFP-β-actin plus Arf4-HA [wild-type (WT), T31N, or Q71L] and analyzed at DIV14 (n = 8–13 neurons). (*B–C*) Quantification of the effect of Arf4 and its mutants on total spine density (*B*) and the densities of specific spine subtypes (*C*) following transfection with Arf4 or its mutants. All data are mean±SEM. * p<0.05, ** p<0.01.

The physiological effects of small GTPases depend on whether they are in a functionally active (GTP-bound) or inactive (GDP-bound) state [30]. To address the effect of Arf4 activity on spine density and morphology, we expressed a series of Arf4 functional mutants in cultured neurons. The Arf4 mutants used included the constitutively active mutant Arf4-Q71L, which remains bound to GTP, and the inactive mutant Arf4-T31N, which remains GDP-bound [36], [46]. Arf4-Q71L had an even more pronounced effect on stimulating spine development than wildtype Arf4, whereas Arf4-T31N did not enhance spine development compared with controls (Figs. 8A and B). Arf4 and its active mutant also promoted stubby and thin spine development (Fig. 8C). Overexpression of the inactive Arf4-T31N mutant significantly reduced mushroom spine density, suggesting that restricting Arf4 to its GDP-bound state prevents the development of mature spines.

### Arf4 is required for normal spine development

**Figure 9:**
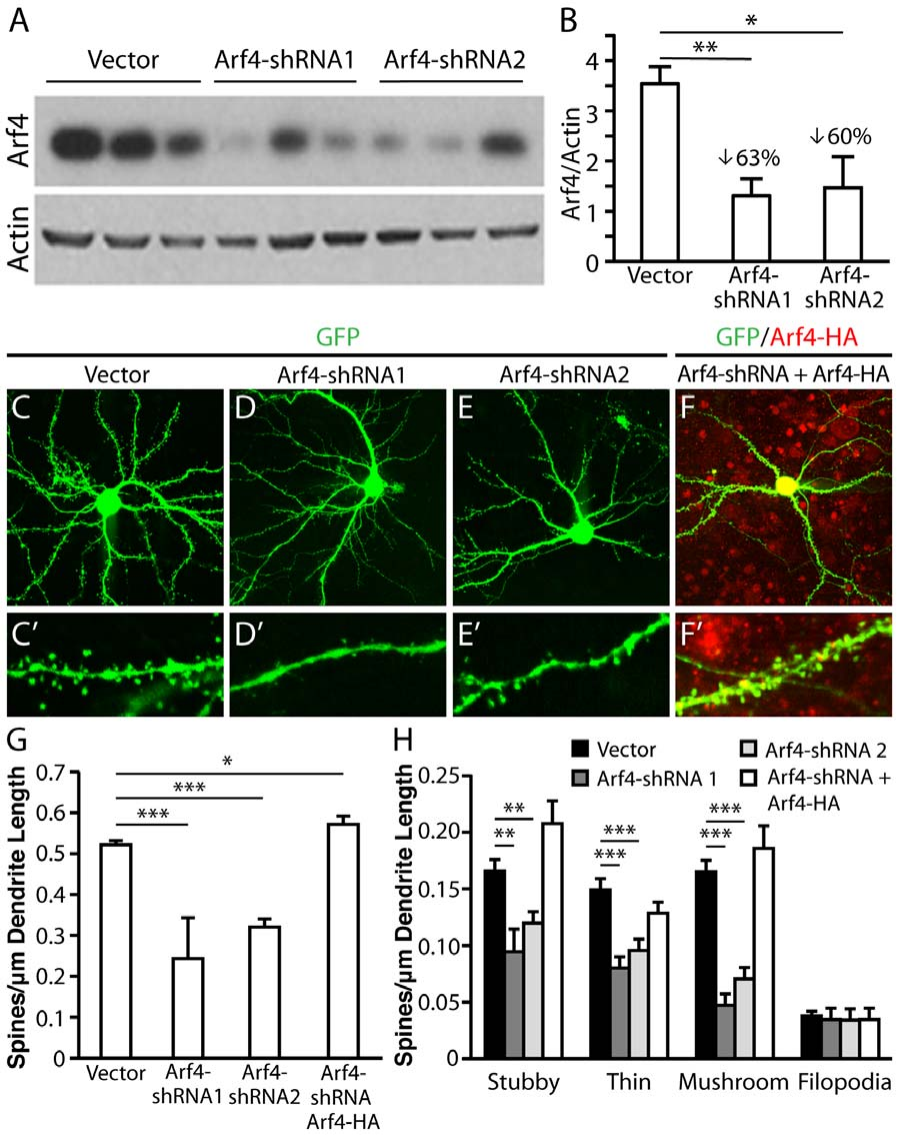
Knockdown of Arf4 by shRNA reduces spine density in mouse primary neurons. (*A*) To verify the efficacy of the Arf4 shRNA-encoding plasmid, mouse Neuro-2A cells were transfected with Arf4-shRNA1 or Arf4-shRNA2 and equal protein amounts of transfected cell lysates were analyzed by immunoblotting with Arf4 antibody, or with actin antibody as a control. (*B*) Quantification of knockdown efficacy by Arf4-shRNA1 or Arf4-shRNA2 in mouse Neuro-2A cells. Arf4 levels are normalized to actin. (*C–F′*) Representative examples of WT neurons transfected with FUGW2-GFP plasmid (*C–C′*), Arf4-shRNA1 (*D–D′*), Arf4-shRNA2 (*E–E′*), or Arf4-shRNA plus Arf4-HA (rescue) (*F–F′*). Lower (*C–F*) and higher (*C′–F′*) magnification images are shown (N = 7–13 neurons per condition). (*G*) Total spine and (*H*) spine subtype densities from each of the experimental conditions. All data are mean±SEM. * p<0.05, ** p<0.01, *** p<0.001.

We next asked whether the absence of endogenous Arf4 perturbs spine development. We transfected neurons at DIV5 with a FUGW2-based vector encoding GFP and one of two mouse Arf4-short hairpin RNA (shRNA) sequences, and then imaged the cells at DIV14. Both Arf4-shRNA1 and Arf4-shRNA2 markedly decreased Arf4 protein levels in Neuro-2a cells 48 hours post-transfection (Figs. 9A and B). Knockdown of Arf4 significantly decreased total spine density (Figs. 9D–E′) as well as the individual densities of all spine subtypes except filopodia (Fig. 9H), compared to controls (Figs. 9C and C′). To verify the target specificity of Arf4-shRNA, we co-expressed human Arf4-HA—whose expression is resistant to mouse shRNA knockdown—with mouse Arf4-shRNA. Expression of human Arf4-HA completely rescued the effects on spine density (Figs. 9F and G) and morphology (Fig. 9H) caused by endogenous mouse Arf4 knockdown, demonstrating the specificity of Arf4-shRNA′s effects.

### ASAP1, an Arf4 GAP, negatively regulates spine development

**Figure 10:**
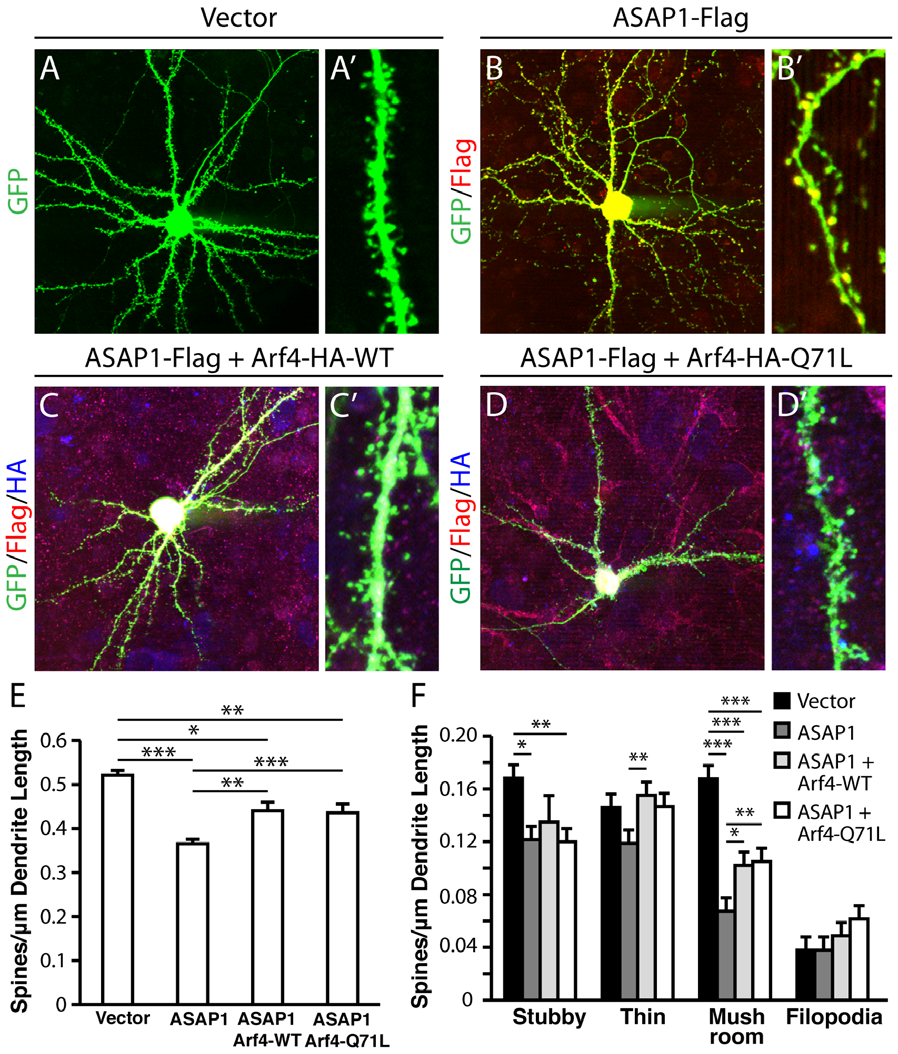
ASAP1, an Arf4 GAP, inhibits dendritic spine formation, and both Arf4-WT and Arf4-Q71L partially rescue this inhibition. (*A−D′*) Representative examples of WT neurons transfected at DIV5 with FUGW2-GFP plasmid alone (*A, A′*), or together with ASAP1-Flag (*B, B′*), ASAP1-Flag plus Arf4-WT-HA (*C, C′*), or ASAP1-Flag plus Arf4-HA Q71L (*D, D′*) and analyzed at DIV14. Lower (*A−D*) and higher (*A′−D′*) magnification images are shown. (n = 11-13 neurons per condition). (*E−F*) Quantification of the effect of ASAP1 alone or together with Arf4-HA-WT or Arf4-HA-Q71L on dendritic spine density (*E*) and morphology (*F*). All data are mean±SEM. * p<0.05, ** p<0.01, *** p<0.001.

Previous studies have shown that ASAP1 functions as an Arf4 GAP and forms a complex with Arf4 [32]. We found that overexpressed ASAP1 is localized to dendrites and dendritic spines in mouse primary neurons, similar to Arf4 (Figs. 10B and B′). Neurons transfected at DIV5 with ASAP1-Flag, together with FUGW2-GFP for visualization of spines, showed a significant decrease in total spine density (Figs. 10B, B′, and E) as well as in stubby and mushroom spine density at DIV14 (Fig. 10F). To determine whether the spine-promoting effect of Arf4 is regulated by ASAP1, we co-transfected primary neurons with ASAP1-Flag and either Arf4-HA-WT or constitutively active Arf4-HA-Q71L, together with FUGW2-GFP. Both WT (Figs. 10C and C′) and constitutively active (Figs. 10D and D′) Arf4 partially blocked ASAP1-induced changes in spine density and morphology, suggesting that ASAP1 is a negative regulator of Arf4′s effects on spine density.

### BDNF levels are not altered in Arf4^+/−^ mice or in Arf4-overexpressing primary neurons

**Figure 11:**
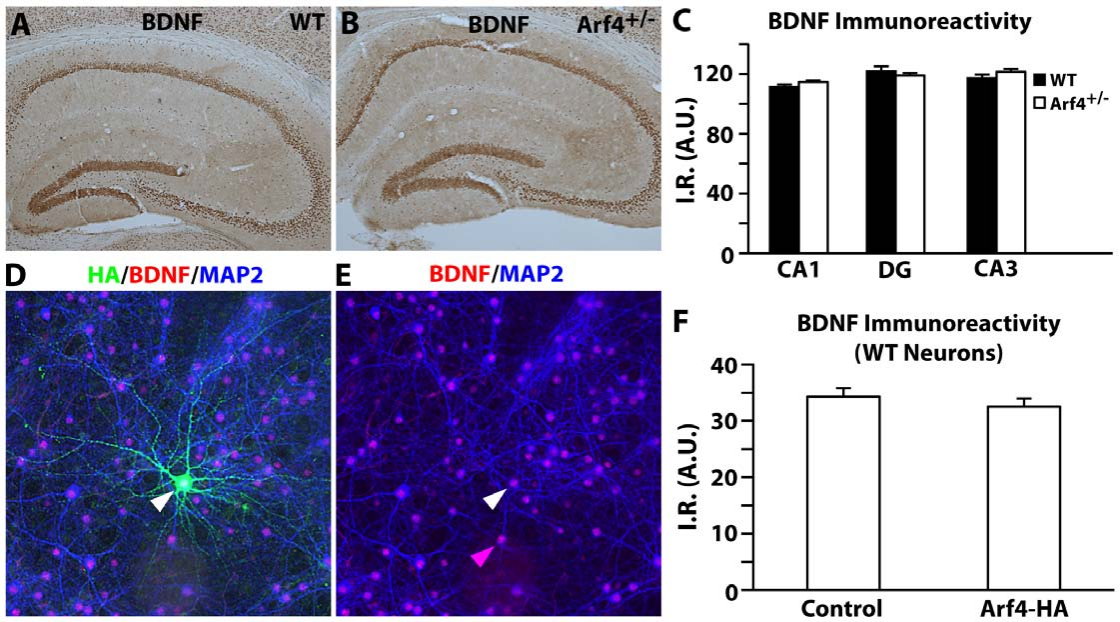
Arf4 levels do not affect BDNF expression *in vivo* or *in vitro*. *(A−B)* Representative images of WT (A) and Arf4^+/−^ (B) hippocampi stained for BDNF, followed by DAB development. *(C)* Quantification of BDNF immunoreactivity in the hippocampus (n = 3 mice per genotype). *(D−E)* Duplicate images of primary neurons transfected with Arf4-HA at DIV5 and stained with HA (green), BDNF (red), and MAP2 (blue) antibodies at DIV14. Arrows point to a neuron overexpressing Arf4-HA (white arrow) or a neuron that does not overexpress Arf4-HA (pink arrow) *(F)* Quantification of BDNF expression in primary neurons either overexpressing or not overexpressing human Arf4-HA (n = 15 neurons per condition). A.U. = arbitrary units.

Since brain-derived neurotrophic factor (BDNF) plays critical roles in both pattern separation [47] and dendritic spine development [48], [49], we asked whether BDNF expression might be altered in Arf4^+/−^ mice. We found that BDNF protein levels in the CA1, CA3, and DG regions of the hippocampus were similar in Arf4^+/−^ mice compared to their wildtype littermates (Fig. 11A–C). BDNF levels also remained unaltered in Arf4-overexpressing neurons compared to neurons not overexpressing Arf4 (Fig. 11D–F). Thus, Arf4 levels do not appear to have a direct effect on neuronal BDNF expression *in vitro* or *in vivo*.

### Arf4 overexpression rescues spine loss in neurons from an AD-related apoE4 mouse model

**Figure 12:**
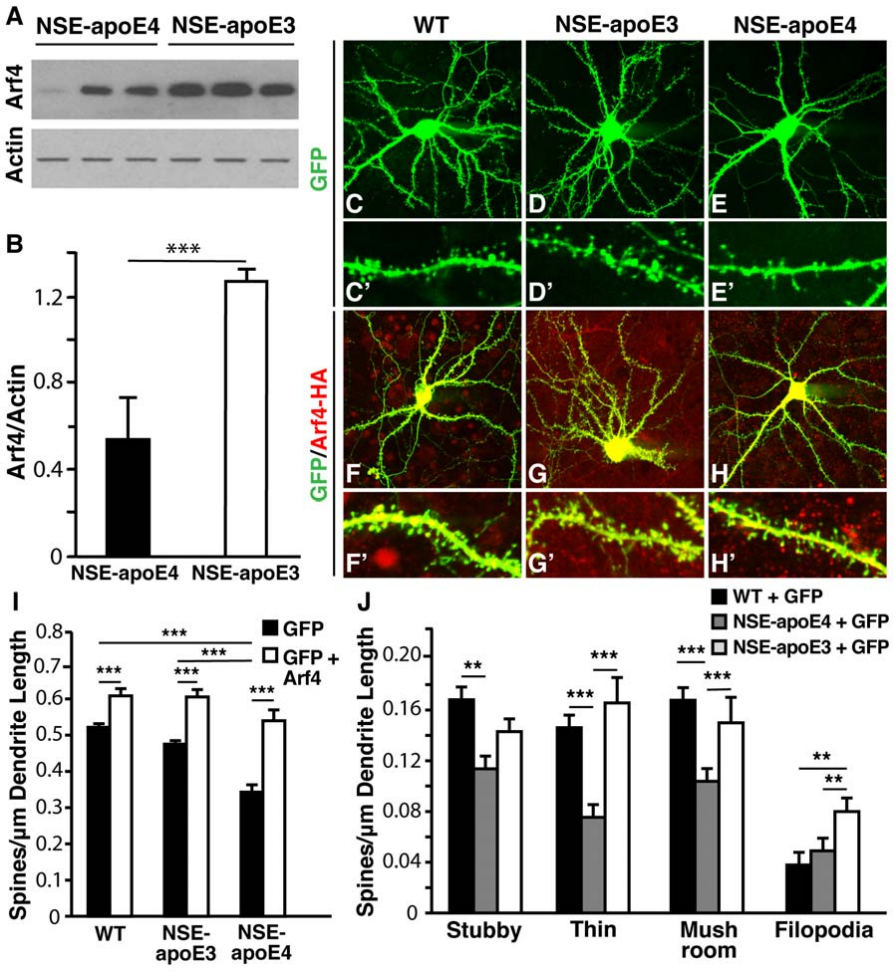
Arf4 overexpression rescues spine loss in apoE4-expressing primary neurons. (*A*) Representative Western blot of actin and Arf4 levels in NSE-apoE4 and -apoE3 mice reveal a decrease in Arf4 protein levels from NSE-apoE4 mice compared to those from NSE-apoE3 mice. Primary neuron lysates were prepared in triplicate, and actin was used as a loading control. (*B*) Quantification of the ratio of Arf4/Actin levels. Data are mean±SD. * p<0.05, *** p<0.001. (*C–H′*) Primary hippocampal and cortical neurons were cultured from WT, homozygous NSE-apoE3, or homozygous NSE-apoE4 mouse E18−E19 embryos. Neurons were transfected with FUGW2 plasmid alone (for visualization of spines) or with FUGW2 plus Arf4-HA-WT at DIV5 and analyzed at DIV14. Lower (*C−H*) and higher (*C′−H′*) magnification images are shown. (*C–E′*) WT (*C–C′*), NSE-apoE3 (*D–D′*), or NSE-apoE4 (*E–E′*) neurons transfected with empty FUGW2 plasmid (n = 12–13 neurons). (*F–H′*) WT (*F–F′*), NSE-apoE3 (*G–G′*), or NSE-apoE4 (*H–H′*) neurons cotransfected with FUGW2 plus Arf4-HA-WT (n = 8–12 neurons). (*I*) Quantification of total spine densities for each experimental condition. (*J*) Spine subtype densities for each experimental condition. All data are mean±SEM. * p<0.05, ** p<0.01, *** p<0.001.

We next examined Arf4′s role in spine development in the context of a neurodegenerative disease model. We investigated the possibility that Arf4 might rescue apoE4-caused spine loss in neurons from transgenic mice expressing apoE4 selectively in neurons [neuron-specific enolase (NSE)-apoE4]. These mice have impairments in learning and memory [50], as well as a loss of dendritic spines in primary neurons and in the hippocampus and cortex [21]. We found that Arf4 mRNA levels were significantly reduced in hippocampi from 10-month-old female NSE-apoE4 (vs. NSE-apoE3) mice (36% reduction, p = 0.015). Furthermore, Arf4 protein levels were also significantly reduced in primary neurons from NSE-apoE4 mice compared to those from NSE-apoE3 mice (Figs. 12A and B).

Consistent with previous findings [21], the dendritic spine density of NSE-apoE4 neurons (Figs. 12E and E′) was significantly less than that of either NSE-apoE3 (Figs. 12D and D′) or WT neurons (Figs. 12C and C′). Additionally, NSE-apoE4 neurons had fewer stubby, thin, and mushroom spines compared with WT neurons (Fig. 12J). Overexpression of Arf4 fully restored the spine loss observed in NSE-apoE4 neurons (Figs. 12H and H′). Furthermore, Arf4 overexpression increased spine density to an extent similar to that observed in Arf4-overexpressing WT (Figs. 12F and F′) and NSE-apoE3 neurons (Figs. 12G and G′). The apoE4-induced decrease in specific spine subtypes was also rescued by Arf4 overexpression (Fig. 12J).

## Discussion

In this study, we show that the small GTPase Arf4 is a novel modulator of DG-dependent pattern separation tasks by regulating dendritic spine development. The loss of one copy of Arf4 *in vivo* leads to severe impairments in pattern separation, as well as a decrease in DG granule cell spine density and mEPSC amplitude. In primary neuron cultures, overexpression of wildtype Arf4 promotes spine development even at an early stage (DIV12), whereas shRNA knockdown of Arf4 inhibits it. These effects are partially mediated by ASAP1, an Arf4 GAP. In addition, overexpression of Arf4 rescues the dendritic spine loss caused by apoE4, the major genetic risk factor for AD. These results indicate that Arf4, by promoting spine development, represents a useful target for treatments of neurodegenerative diseases that cause profound synaptic loss, such as AD.

### Arf4 regulates dendritic spine density and morphology

Spine number and structural plasticity are tightly correlated with synaptic function in the mammalian brain [23], [51]. We found that Arf4 overexpression promotes spine development, particularly that of thin spines. Previous studies have shown that thin spines are more transient and motile than mushroom spines, and an increase in the proportion of thin spines represents a greater capacity to stabilize after LTP [52], [53]. Mushroom spines have larger postsynaptic densities (PSDs) and more glutamate receptors than thin spines, making the synapse functionally stronger and more stable [14], [23]. We found that knocking down Arf4 decreases thin and mushroom spine density and, consistently, impairs the electrophysiological function of granule cells of the dentate gyrus. Decreases in both spine types have been reported in animal models of cognitive decline [54], [55]. Based on these results, a significant loss of both transient thin spines and stable mushroom spines in primary neurons lacking Arf4 raises the possibility that Arf4 might be critical for both dendritic spine plasticity and stability.

Our study uncovered potential molecular mechanisms governing the effects of Arf4 on spine density and morphology. First, we found that the effects of Arf4 on spine development are related to its activity state. The GTP-bound form of Arf4 stably associates with intracellular organelle membranes and can activate downstream effectors [56], [46]. By constitutively binding to GTP, Arf4-Q71L likely activates signaling pathways that can induce spine morphogenesis to a greater extent than wildtype Arf4. In contrast, Arf4-T31N remains bound to GDP and is therefore theoretically inactive. Previous studies have reported seemingly contradictory roles for GDP-bound Arf proteins (Arf-T31N) as either inactive mutants whose physiological effects do not differ from wildtype Arfs [57], [58], or as dominant-negative mutants with inhibitory effects on vesicular transport and other cellular functions [59]. Interestingly, our results suggest that Arf4-T31N acts as an inactive mutant with respect to its effects on total spine density, but displays some dominant-negative characteristics by specifically reducing the density of mushroom spines.

Small GTPases modulate actin cytoskeletal rearrangement and dynamics in both neuronal and non-neuronal cells [10], [60], suggesting that actin-binding proteins or their regulators might serve as effectors of Arf4 in spine morphogenesis. We found that the Arf GAP ASAP1 negatively regulates dendritic spine density compared to controls, and this effect is partially rescued by co-expression of ASAP1 together with Arf4-WT. ASAP1 is known to regulate the actin cytoskeleton [61], indicating that Arf4 and ASAP1 might interact to influence spine cytoskeletal dynamics. Recent studies have shown that Arf activation promotes recruitment of several actin-regulatory proteins, including cortactin and dynamin, to the vesicle-budding sites of the trans-Golgi network (TGN) [62], [63]. Our finding that Arf4 colocalizes with actin in primary neurons suggests a potential interplay between Arf4 and actin-regulating proteins in dendritic spines.

### Role of Arf4 in dentate gyrus-dependent pattern separation tasks

Mossy fiber “detonator” synapses arising from DG granule cells strongly activate the CA3 region and are involved in a variety of neurological functions, including memory and spatial representations [6], [64]. Our experiments showed that Arf4 heterozygosity *in vivo* leads to reductions in spine number and mEPSC amplitude in the DG, and these alterations accompany profound pattern separation impairments. These results indicate that Arf4 could serve as a novel regulator of the mossy fiber pathway by regulating granule cell spine development and electrophysiological activity.

Spine head size is positively correlated with mEPSC amplitude [65], and the functional loss of NMDA receptors in DG granule cells has been associated with impairments in pattern separation [8]. In our study, mushroom spine numbers were significantly lower in the DG of Arf4^+/−^ compared to WT mice, and this spine loss correlated with a reduction in mEPSC amplitude. Mushroom spines contain more AMPA and NMDA receptors than other spine types, and these receptors are critical for strengthening synaptic connections [14]. Therefore, the context recognition impairments seen in Arf4^+/−^ mice could be related to reduced granule cell-specific synaptic communication caused by the loss of mushroom-type spines.

### Arf4 overexpression as a potential therapeutic strategy for AD-related spine loss

As spine loss is strongly correlated with cognitive impairments in AD [15], [66], a potential therapeutic strategy for restoring cognitive function in AD patients could be to increase dendritic spine density, thereby strengthening synaptic connections. In our current study, we found that overexpression of Arf4 restored spine loss in NSE-apoE4 neurons. Thus, increasing Arf4′s function might serve as a potential therapeutic strategy for restoring impairments in spine development and synaptic connectivity.

The preclinical period prior to the diagnosis of AD is characterized by deficits in a number of memory-related processes, including pattern separation [64], [67]. Patients with amnestic mild cognitive impairment (MCI), for instance, showed an impaired ability to distinguish between a previously seen object and a very similar but unobserved object [68]. Some aged rodents that do not develop AD pathology nevertheless have memory deficits, such as a failure to encode novel information while navigating similar situations [69]. In our current study, it is intriguing that young (4–5 month old) Arf4^+/−^ mice have pattern separation deficits that are similar to those found in pre-clinical MCI patients and aged rodents, but exhibit normal spatial learning and memory. The Arf4^+/−^ mouse model might therefore represent a unique tool to investigate early cognitive dysfunctions prior to the onset of AD pathology. Our studies should provide further understanding of the molecular mechanisms underlying DG-dependent memory tasks and spine development, and how these processes are degraded in neurodegenerative disorders, such as AD.
